# Computational codon optimization of synthetic gene for protein expression

**DOI:** 10.1186/1752-0509-6-134

**Published:** 2012-10-20

**Authors:** Bevan Kai-Sheng Chung, Dong-Yup Lee

**Affiliations:** 1 Department of Chemical and Biomolecular Engineering, National University of Singapore, 4 Engineering Drive 4, Singapore, 117576, Singapore; 2 NUS Graduate School for Integrative Sciences and Engineering, National University of Singapore, 28 Medical Drive, #05-01, Singapore, 117456, Singapore; 3 Bioprocessing Technology Institute, Agency for Science, Technology and Research (A*STAR), 20 Biopolis Way, #06-01 Centros, Singapore, 138668, Singapore

## Abstract

**Background:**

The construction of customized nucleic acid sequences allows us to have greater flexibility in gene design for recombinant protein expression. Among the various parameters considered for such DNA sequence design, individual codon usage (ICU) has been implicated as one of the most crucial factors affecting mRNA translational efficiency. However, previous works have also reported the significant influence of codon pair usage, also known as codon context (CC), on the level of protein expression.

**Results:**

In this study, we have developed novel computational procedures for evaluating the relative importance of optimizing ICU and CC for enhancing protein expression. By formulating appropriate mathematical expressions to quantify the ICU and CC fitness of a coding sequence, optimization procedures based on genetic algorithm were employed to maximize its ICU and/or CC fitness. Surprisingly, the *in silico* validation of the resultant optimized DNA sequences for *Escherichia coli*, *Lactococcus lactis*, *Pichia pastoris* and *Saccharomyces cerevisiae* suggests that CC is a more relevant design criterion than the commonly considered ICU.

**Conclusions:**

The proposed CC optimization framework can complement and enhance the capabilities of current gene design tools, with potential applications to heterologous protein production and even vaccine development in synthetic biotechnology.

## Background

Recent developments in artificial gene synthesis have enabled the construction of synthetic gene circuits [1] and even the synthesis of whole bacterial genome [2]. The introduction of synthetic genes into a living system can either modulate existing biological functions or give rise to novel cellular behavior. In this sense, de novo gene synthesis is a valuable synthetic biological tool for biotechnological studies, which typically aims to improve tolerance to toxic molecules, retrofit existing biosynthetic pathways, design novel biosynthetic pathways and/or enhance heterologous protein production [3,4]. In the aspect of recombinant protein production, natural genes found in wild-type organisms are usually transformed into the heterologous hosts for recombinant expression. This approach typically results in poorly expressed recombinant protein since the wild-type foreign genes have not been evolved for optimum expression in the host. Thus, it is highly desirable to harness the flexibility in synthetic biology to create customized artificial gene designs that are optimal for heterologous protein expression. To aid the gene design process, computational tools have been developed for designing coding sequences based on some performance criteria.

Specifically, the degeneracy of the genetic code, reflected by the use of sixty-four codons to encode twenty amino acids and translation termination signal, leads to the situation whereby all amino acids, except methionine and tryptophan, can be encoded by two to six synonymous codons. Notably, the synonymous codons are not equally utilized to encode the amino acids, thus resulting in phenomenon of codon usage bias which was first reported in a study that examines the frequencies of 61 amino acid codons (i.e. termination codons are excluded) in 90 genes [5]. The emergence of codon usage bias in organisms has been largely attributed to natural selection, mutation, and genetic drift [6]. More importantly, codon usage bias has been shown to be correlated to gene expression level [7,8]. As a result, this bias has been proposed as an important design parameter for enhancing recombinant protein production in heterologous expression hosts [9]. Consequently, the algorithms implemented in many of the sequence design software tools, such as Codon optimizer [10], Gene Designer [11], and OPTIMIZER [12], are mainly focused on the frequency of individual codon occurrences. Notably, the popular web-based software, known as the Java Codon Adaptation Tool (JCat), is integrated with the PRODORIC database to allow convenient retrieval of prokaryotic genetic information [13,14]. However, apart from individual codon usage (ICU) bias, nonrandom utilization of adjacent codon pairs in organisms has also been reported in several studies [15,16]. This phenomenon is termed “codon context” as it implicates some “rule” for organizing neighboring codons as a result of potential tRNA-tRNA steric interaction within the ribosomes [17,18]. Codon context (CC) was shown to correlate with translation elongation rate such that the usage of rare codon pairs decreased protein translation rates [19]. Therefore, the incorporation of CC has been proposed in the conventional ICU-based gene optimization algorithm GeneOptimizer [20]. Furthermore, a patented technology, known as “Translation Engineering”, demonstrated that better enhancement in translational efficiency is achievable by optimizing codon pair usage in addition to ICU optimization [21]. However, there is yet a study to investigate the relative effects of ICU and CC on protein expression. To address this issue, we propose a computational analysis to evaluate the performance of sequences generated by various ICU and CC optimization approaches.

In this study, we applied novel computational procedures to generate DNA sequences exhibiting optimal ICU and CC in *Escherichia coli*, *Lactococcus lactis*, *Pichia pastoris* and *Saccharomyces cerevisiae* based on information obtained from omics data analysis. While *E. coli* and *S. cerevisiae* has been model organisms for recombinant protein production studies, we also consider codon optimization in the Gram-positive bacterium *L. lactis* and methylotrophic yeast *P. pastoris* since they are also promising candidates for expressing recombinant proteins [22,23]. Assuming that the native DNA sequences of highly expressed genes have evolved to exhibit optimal ICU and CC for high *in vivo* expression, we demonstrated the efficacy of our computational approaches by performing a leave-one-out cross-validation on the high-expression genes for each expression host.

## Results

### Codon optimization formulation

To investigate the relative importance of ICU and CC towards designing sequences for high protein expression, we implemented three computational procedures: the individual codon usage optimization (ICO) method generates a sequence with optimal ICU only; the codon context optimization (CCO) method optimizes sequences with regard to codon context only; and the multi-objective codon optimization (MOCO) method simultaneously considers both ICU and CC. Thus, the resultant sequence is ICU-/CC-optimal when its ICU/CC distribution is closest to the organism’s reference ICU/CC distribution calculated based on the sequences of native high-expression genes. Based on the mathematical formulation presented in Methods, the ICO problem can be described as the maximization of ICU fitness, *Ψ*_ICU_ (see Eqn. 23), subject to the constraint that the codon sequence can be translated into the target protein (see Eqns. 3, 4 and 11). Due to the discrete codon variables and nonlinear fitness expression of *Ψ*_ICU_, ICO is classified a mixed-integer nonlinear programming (MINLP) problem. Nonetheless, it can be linearized using a strategy shown in an earlier study by decomposing the nonlinear |*p*_0_^*k*^ − *p*_1_^*k*^| term (see Equation 23) into a series of linear and integer constraints which consist of binary and positive real variables [24]. The resultant mixed-integer linear programming (MILP) problem can be solved using well established computational methods such as either branch-and-bound and branch-and-cut [25]. However, due to the large and discrete search space which contains all possible DNA sequences that can encode the target protein, solving the MILP using these methods may require a long computational time. Thus, alternative methods, such as GASCO [26] and QPSOBT [27], have been proposed for solving ICO using genetic algorithm and particle swarm optimization. Although these heuristic methods are more efficient than conventional MILP solving procedures, they still require a significant amount of computational resources due to the iterative nature of the algorithms. To circumvent the high computational costs, we developed the non-iterative method for solving ICO using the following steps:

I1. Calculate the host’s individual codon usage distribution, *p*_0_^*k*^.

I2. Calculate the subject’s amino acid counts, *θ*_AA,1_^*j*^.

I3. Calculate the optimal codon counts for the subject using the expression: $\theta_{\text{C,opt}}^{k}=p_{0}^{k}\times \sum_{j=1}^{21}\left[\theta_{\text{A},1}^{j}\times 1\left\{\alpha^{j}=f\left(\kappa^{k}\right)\right\}\right]\;\forall k\in \left\{1,2,\ldots ,64\right\}$.

I4. For each *τ*_*i*_ in the subject’s sequence, randomly assign a codon *κ*^*k*^ if *θ*_C_^*k*^ > 0, and decrement *θ*_C,opt_^*k*^ by one.

I5. Repeat step I4 for all amino acids of the target protein from *τ*_1,1_ to *τ*_*n*,1_.

Similarly, CCO can be formulated as the maximization of CC fitness, *Ψ*_CC_ (see Eqn. 26), subject to the constraint that the codon pair sequence can be translated into the target protein (see Eqns. 7, 8 and 12). To find the solution for CCO, the procedure in ICO may not be applicable due to the computational complexity which arises from the dependency of adjacent codon pairs. For example, given a codon pair “AUG-AGA” in a 5’-3’ direction, the following codon pair must only start with “AGA”. Therefore, if we had adopted the ICO procedure to directly identify the codon pairs and randomly assign them to the respective amino acid pairs, there could be conflicting codon pair assignments in certain parts of the sequence. Since the characteristic of independency, which was exploited to develop a simple solution procedure for ICO, is absent in the CCO problem, we resort to a more sophisticated computational approach.

The CCO problem can be conceptualized in a similar way as the well-known traveling salesman problem whereby the traversing from one codon to the next adjacent codon is analogous to the salesman traveling from one city to the next [28]. Since there will be a “cost” incurred by taking a particular “codon path”, the CCO problem aims to minimize of the total cost for traveling a codon path that is able to code the desired protein sequence. However, the CCO problem is more complex than the traveling salesman problem due to the nonlinear cost function evaluated based on the frequency of codon pair occurrence (see Materials and Methods). For an average sized protein consisting 300 amino acids, the total number of codon paths can be as many as 10^100^. Finding an optimal solution for such a large-scale combinatorial problem within an acceptable period of computation time can only be achieved via heuristic optimization methods. Incidentally, the use of genetic algorithm [29] provides an intuitive framework whereby codon path candidates are “evolved” towards optimal CC through techniques mimicking natural evolutionary processes such as selection, crossover or recombination and mutation. Thus, the procedure for solving CCO is as follows:

C1. Randomly initialize a population of coding sequences for target protein.

C2. Evaluate the CC fitness of each sequence in the population.

C3. Rank the sequences by CC fitness and check termination criterion.

C4. If termination criterion is not satisfied, select the “fittest” sequences (top 50% of the population) as the parents for creation of offsprings via recombination and mutation.

C5. Combine the parents and offsprings to form a new population.

C6. Repeat steps C2 to C5 until termination criterion is satisfied.

In step C3, the termination criterion depends on the degree of improvement in best CC fitness values for consecutive generations of the genetic algorithm. If the improvement in CC fitness across many generations is not significant, the algorithm is said to have converged. In this study, the CC optimization algorithm is set to terminate when there is less than 0.5% increase in CC fitness across 100 generations, i.e. *Ψ*_CC_^(*r* + 100)^/*Ψ*_CC_^(*r*)^ < 0.005 where *r* refers to the *r*^th^ generation of the genetic algorithm. When the termination criterion is not satisfied, the subsequent step C4 will perform an elitist selection such that the fittest 50% of the population are always selected for reproduction of offsprings through recombination and mutation. During recombination, a pair of parents is chosen at random and a crossover is carried out at a randomly selected position in the parents’ sequences to create 2 new individuals as offsprings. The offsprings subsequently undergo a random point mutation before they are combined with the parents to form the new generation.

Unlike traditional implementations of genetic algorithm where individuals in the population are represented as as 0–1 bit strings, the presented CC optimization algorithm represents each individual as a sequential list of character triplets indicating the respective codons. Therefore, the codons can be manipulated directly with reference to a hash table which defines the synonymous codons for each amino acid. As a result, the protein encoded by the coding sequences is always the same in the genetic algorithm since crossovers only occur at the boundary of the codon triplets and mutation is always performed with reference to the hash table of synonymous codons for each respective amino acid.

Based on the formulations for ICU and CC optimization, the MOCO problem, which is an integration of both, can be described as maximizing both ICU and CC fitness, i.e. max (*Ψ*_ICU_*Ψ*_CC_), subject to the constraints that both the codon and codon pair sequences can be translated into the target protein sequence. As such, due to the complexity attributed to CC optimization, solution to MOCO will also require a heuristic method. In this case, the nondominated sorting genetic algorithm-II (NSGA-II) is used to solve the multi-objective optimization problem [30]. The procedure for NSGA-II is similar to that presented for CC optimization except for additional steps required to identify the nondominated solution sets and the ranking of these sets to identify the pareto optimum front. The NSGA-II procedure for solving the MOCO problem is as follows:

M1. Randomly initialize a population of coding sequences for target protein.

M2. Evaluate ICU and CC fitness of each sequence in the population.

M3. Group the sequences into nondominated sets and rank the sets.

M4. Check termination criterion.

M5. If termination criterion is not satisfied, select the “fittest” sequences (top 50% of the population) as the parents for creation of offsprings via recombination and mutation.

M6. Combine the parents and offsprings to form a new population.

M7. Repeat steps M2 to M5 until termination criterion is satisfied.

The identification and ranking of nondominated sets in step M3 is performed via pair-wise comparison of the sequences’ ICU and CC fitness. For a given pair of sequences with fitness values expressed as (*Ψ*_ICU_^1^, *Ψ*_CC_^1^) and (*Ψ*_ICU_^2^, *Ψ*_CC_^2^), the domination status can be evaluated using the following rules:

• If (*Ψ*_ICU_^1^ > *Ψ*_ICU_^2^) and (*Ψ*_CC_^1^ ≥ *Ψ*_CC_^2^), sequence 1 dominates sequence 2.

• If (*Ψ*_ICU_^1^ ≥ *Ψ*_ICU_^2^) and (*Ψ*_CC_^1^ > *Ψ*_CC_^2^), sequence 1 dominates sequence 2.

• If (*Ψ*_ICU_^1^ < *Ψ*_ICU_^2^) and (*Ψ*_CC_^1^ ≤ *Ψ*_CC_^2^), sequence 2 dominates sequence 1.

• If (*Ψ*_ICU_^1^ ≤ *Ψ*_ICU_^2^) and (*Ψ*_CC_^1^ < *Ψ*_CC_^2^), sequence 2 dominates sequence 1.

**Figure 1:**
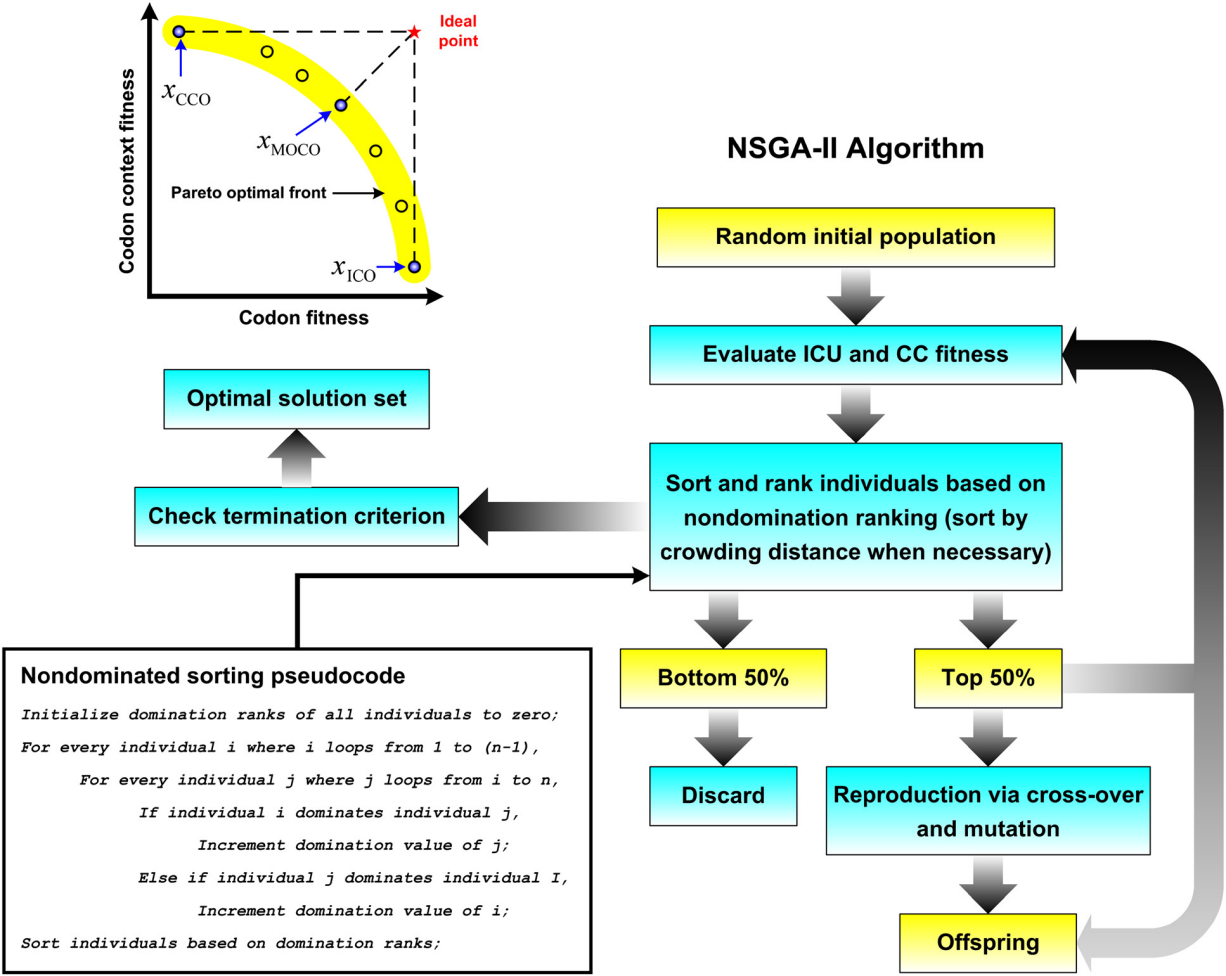
**Multi-objective codon optimization solution.** The optimal solutions generated by MOCO lies on the pareto front (region in yellow).

Whenever a particular sequence is found to be dominated by another sequence, the domination rank of the former sequence is lowered. As such, the grouping and sorting of the nondominated sets are performed simultaneously in step M3 (Figure 1). In the original nondominated sorting algorithm [30], the set of individuals that is dominated by every individual is stored in memory. Therefore, for a total population of *n*, the total storage requirement is *O*(*n*^2^). However, for the abovementioned algorithm, only *O*(*n*) storage is required for storing the domination value of each individual. In terms of computational complexity, both the original and modified algorithm requires at most *O*(*mn*^2^) computations for *m* objective values since all the *n* individuals have to be compared pair-wise for every objective to be optimized. Therefore, the nondominated sorting algorithm presented in this thesis is superior on the whole, especially with regards to computational storage requirement which can become an important issue when dealing with long coding sequences.

The output of multi-objective optimization is a set of solutions also known as the pareto optimal front. Since the aim of MOCO is to examine the relative effects of ICU and CC optimization, it is not necessary to analyze all the sequences in the pareto optimal front. Instead, the solution which is nearest to the ideal point will represent the sequence with balanced ICU and CC optimality. As such, the solutions of ICO, CCO and MOCO will subsequently be referred to as *x*_ICO_, *x*_CCO_ and *x*_MOCO_ respectively (Figure 1). The program for performing ICO, CCO and MOCO can be downloaded from the following link: http://bioinfo.bti.a-star.edu.sg/tool/CodonOptimization/.

### Finding the codon preference

**Figure 2:**
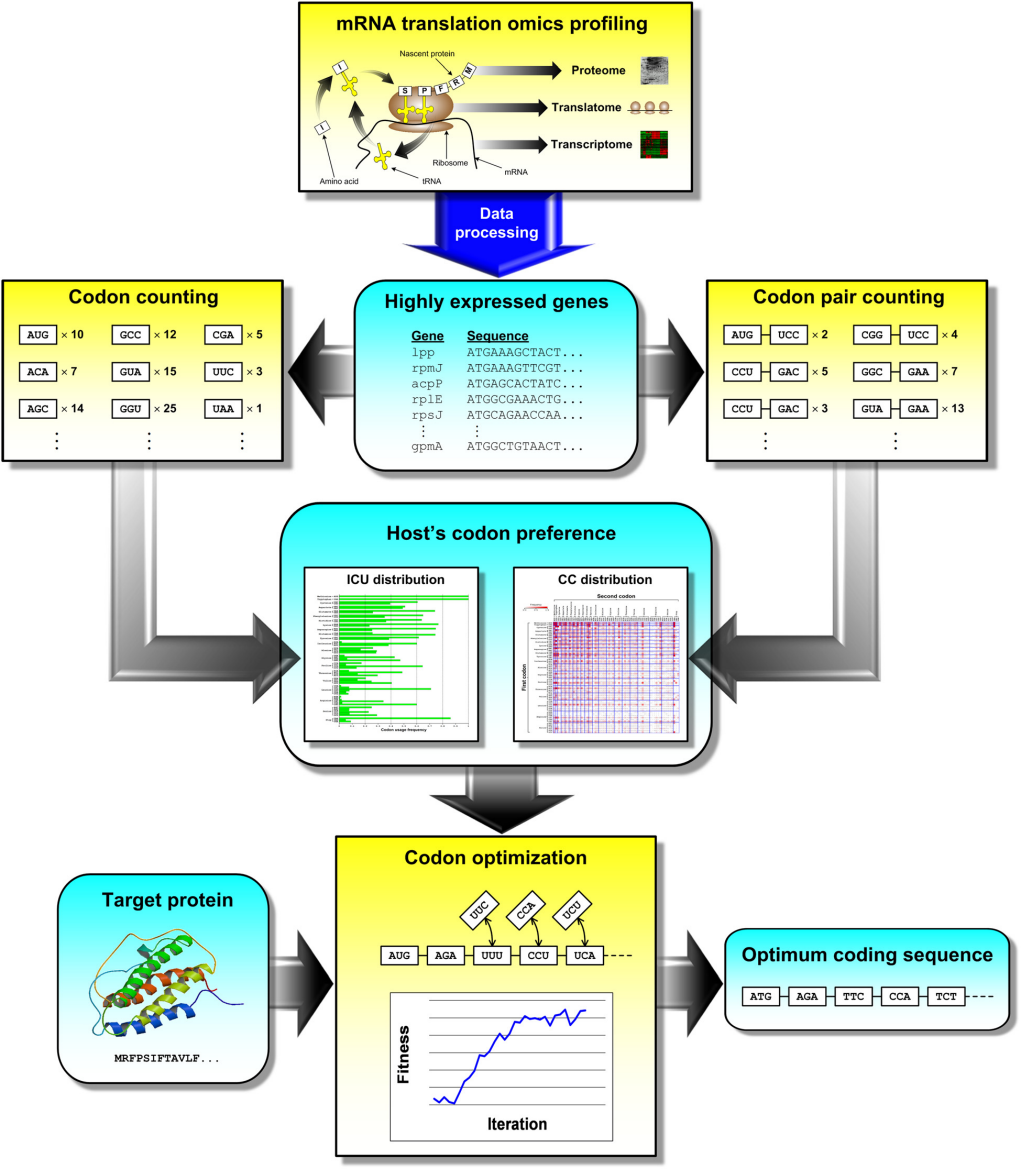
**General codon optimization workflow.** In the step of codon optimization, either ICO, CCO or MOCO can be used to optimized the sequence.

The entire workflow for codon optimization of a target protein sequence begins with the identification of the host’s preferred ICU and CC distributions as the reference (Figure 2). These ICU and CC distributions should ideally capture codon usage patterns that correspond to efficient translation of mRNA to protein. Therefore, the first step of codon optimization identifies the reference ICU and CC distributions by characterizing the underlying mechanisms of efficient translation which can be achieved through transcriptome, translatome and proteome profiling as demonstrated in earlier studies [31,32]. However, such large-scale experimental data are not readily available for the extraction of codon usage preference information in all the expression hosts considered in this study. Alternatively, it is assumed that the organisms have evolved to conserve resources by producing high amounts of transcripts for genes that will also be efficiently translated. As such, the widely available transcriptome data from microarray experiments can be used to identify the highly expressed and efficiently translated genes. Thus, the codon pattern of the host’s native high-expression genes will be a suitable reference point for codon optimization.

The step for selecting high-expression genes codon pattern for codon optimization is only relevant if the following two conditions are true: (1) ICU and CC distributions of high-expression genes are significantly biased and nonrandom; and (2) there is a significant difference in ICU and CC distribution between highly expressed genes and all the genes in the host organism’s genome. It is noted that if the first condition is false, there is no codon (pair) bias and codons can be assigned randomly based on a uniform distribution; if the second condition is false, the computation of ICU and CC distributions based on all the genes in the genome will be sufficient to characterize the ICU and CC preference of the organism without the need for selecting high-expression genes.

**Figure 3:**
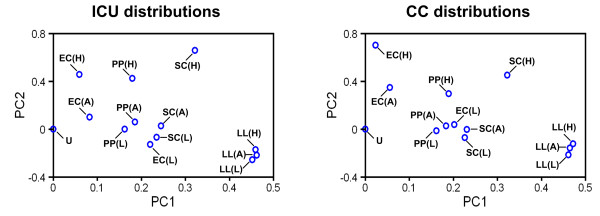
**PCA of ICU and CC distributions.** The first and second principal components (PC1 and PC2) are plotted to show the differences in the ICU and CC distributions of (top 5%) high-expression genes (H), (bottom 5%) low-expression genes (L) and all genes (A) found in the genomes of *E. coli* (EC), *L. lactis* (LL), *P. pastoris* (PP) and *S. cerevisiae* (SC). The unbiased distribution (U) is also included for each plot as reference.

To determine the significance of ICU and CC biases, we applied the Pearson’s chi-squared test (see Materials and Methods). Using a p-value cut-off of 0.05, the ICU and CC distributions of at least 80% of the amino acids (pairs) amenable to the chi-squared test were found to be significantly biased in the micro-organisms (Table 1). In the high-expression genes, aspartate was found to be the only one among all amino acids exhibiting an ICU distribution that is not significantly different from the unbiased distribution for *E. coli*, *P. pastoris* and *S. cerevisiae*. Similarly, more than 80% of the amino acids (pairs) show significant difference in ICU and CC distributions between high-expression genes and all genes in the genomes of these three microbes. Contrastingly, 80% amino acids did not show significant difference in CC distributions between high-expression genes and all genes in *L. lactis*, suggesting that the selection of highly expressed genes may not be required to establish the CC preference of *L. lactis*. By applying the principal component analysis, we can observe that the ICU and CC distributions for all types of genes in *L. lactis* are close to one another when compared to genes from other organisms (Figure 3). This indicates that the short listing of highly expressed genes may not be necessary for organisms like *L. lactis*. Nonetheless, we recommend the identification of high-expression genes to characterize the ICU and CC preference of any host, such that there is a better level of confidence that the optimized recombinant gene can be efficiently expressed.

**Table 1:** ICU and CC biasness analysis

	***E. coli***		***L. lactis***		***P. pastoris***		***S. cerevisiae***	
Null hypothesis (*H*_0_)	*D*^*H*^ = *U*	*D*^*H*^ = *D*^*A*^	*D*^*H*^ = *U*	*D*^*H*^ = *D*^*A*^	*D*^*H*^ = *U*	*D*^*H*^ = *D*^*A*^	*D*^*H*^ = *U*	*D*^*H*^ = *D*^*A*^
Alternative hypothesis (*H*_1_)	*D*^*H*^ ≠ *U*	*D*^*H*^ ≠ *D*^*A*^	*D*^*H*^ ≠ *U*	*D*^*H*^ ≠ *D*^*A*^	*D*^*H*^ ≠ *U*	*D*^*H*^ ≠ *D*^*A*^	*D*^*H*^ ≠ *U*	*D*^*H*^ ≠ *D*^*A*^
No. of biased amino acids (P-value < 0.05)	18	17	19	17	18	19	18	19
No. of unbiased amino acids (P-value ≥ 0.05)	1	2	0	2	1	0	1	0
No. of singular amino acids	2	2	2	2	2	2	2	2
No. of unevaluated amino acids (Expect count < 5)	0	0	0	0	0	0	0	0
Total no. of amino acids	21	21	21	21	21	21	21	21
No. of biased amino acid pairs (P-value < 0.05)	314	99	327	15	354	259	372	282
No. of unbiased amino acid pairs (P-value ≥ 0.05)	26	23	12	65	38	36	19	9
No. of singular amino acid pairs	4	4	4	4	4	4	4	4
No. of unevaluated amino acid pairs (Expect count < 5)	76	294	77	336	24	121	25	125
Total no. of amino acid pairs	420	420	420	420	420	420	420	420

The chi-squared statistic is computed based on the observed occurrence of each codon (pair) and the expected occurrence under the null hypothesis of uniform distribution. Any amino acid (pair) with p-value < 0.05 is considered to exhibit significantly biased codon (pair) usage. Singular amino acids (methionine and tryptophan) and singular amino acid pairs (pairs only consisting of methionine and/or tryptophan) are not amenable to the biasness analysis since they are not encoded by more than one synonymous codon (pair). Chi-squared statistic and p-value are not calculated for amino acid (pair) with expected counts less than 5 (see Materials and Methods for details). Abbreviations: *D*^*A*^, codon (pair) distribution of all genes in the genome; *D*^*H*^, codon (pair) distribution of high-expression genes; *U*, uniform distribution.

### Performance of codon optimization methods

**Figure 4:**
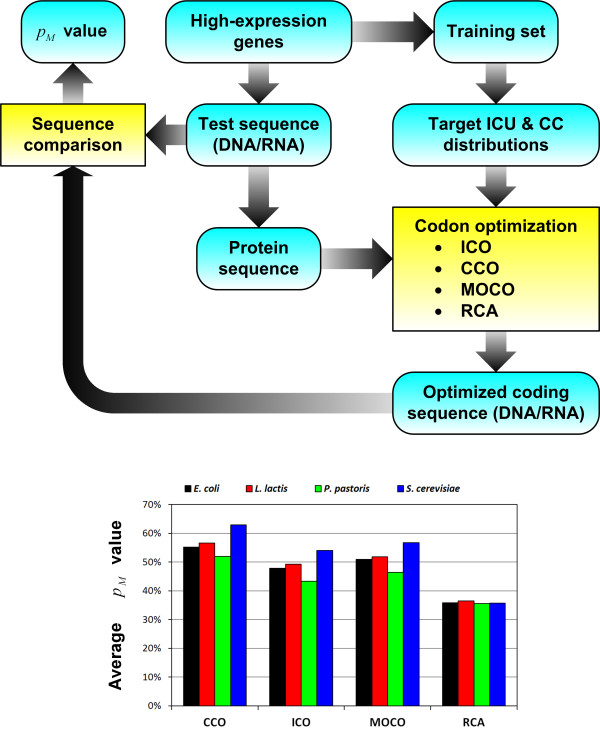
**Codon optimization validation.** The *in silico* cross-validation of the optimization procedures is performed according to the presented workflow.

The performance of each optimization approach was evaluated using a leave-one-out cross-validation, where a gene is randomly selected from the entire set of high-expression genes for sequence optimization while the rest of the genes will be used as the training set to calculate the reference ICU and CC distribution (Figure 4). The predicted optimum sequences are compared with the original native sequences to evaluate the performance of each codon optimization approach (see Additional file 1 for the sequences of the wild-type and optimized genes). As the degree of similarity to the wild-type high expression genes indicates the gene expressivity potential of the optimized sequences, the quality of each optimized sequence was measured in terms of the percentage of codons matching the corresponding native sequence, denoted by *P*_*M*_. From the results, the *x*_ICO_, *x*_CCO_ and *x*_MOCO_ solutions were generally found to be more similar to the native genes than the random sequences generated by RCA indicating that all the optimization approaches are indeed capable of improving the codon usage pattern compared to the control (Figure 4). The *P*_*M*_ values of *x*_ICO_, *x*_CCO_, *x*_MOCO_ and *x*_RCA_ sequences for each gene are further compared in a “tournament” style to show the relative performance of each optimization method. In the tournament matrix (Table 2), each cell shows the number of wins by the method in the left-most column against that in the upper-most row. Whenever the numbers of wins and losses (i.e. cells diagonally opposite of each other) do not sum up to 100, the shortfall will be equal to the number of draws.

**Table 2:** Tournament matrix

	***x***_**ICO**_	***x***_**CCO**_	***x***_**MOCO**_	***x***_**RCA**_
		7	19	95
		2	18	99
*x*_ICO_		4	15	93
		5	22	99
	92		82	97
	96		93	100
*x*_CCO_	96		86	100
	93		89	99
	78	15		97
	74	5		100
*x*_MOCO_	83	12		99
	75	9		99
	5	2	3	
	0	0	0	
*x*_RCA_	6	0	1	
	1	0	0	

For every gene, the *p*_*M*_ of the optimal sequences generated by respective optimization approaches are compared pair-wise for each expression host. The numbers of tournament wins/losses by each approach for all the genes in each expression host are added up. The sequences generated by ICO, CCO, MOCO and RCA are indicated as *x*_ICO_, *x*_CCO_, *x*_MOCO_ and *x*_RCA_ respectively. In each cell, the numbers from top-most to bottom-most corresponds to the data for *E. coli*, *L. lactis*, *P. pastoris* and *S. cerevisiae*, respectively.

Through the comparison of ICO and CCO, the *x*_CCO_ solutions have a higher average percentage of codon matches than *x*_ICO_ sequences for all four microbes (Figure 4), with at least 90% of the *x*_CCO_ sequences matching the native corresponding sequences better than those generated by ICO (Table 2). This result indicates that CC fitness can be a more important design parameter for sequence optimization than ICU fitness which has been a conventional design criterion implemented in several software tools. While it appears likely that the integration of CCO with ICO under a multi-objective optimization framework can potentially lead to even better sequence design, results from our MOCO analysis suggest otherwise. The average of *P*_*M*_ value of *x*_MOCO_ were observed to be lower than that of *x*_CCO_, indicating that the consideration of ICU fitness in addition to CC fitness can be detrimental to the sequence design. To our best knowledge, no such formal evaluation of the relative impact of ICU and CC fitness on synthetic gene design has been presented to date. Hence, based on the promising *in silico* validation results which implicate CC as an important design parameter for optimizing sequences, the newly developed CCO procedure can potentially supersede the ICU optimization techniques currently implemented in gene design software tools. It is noted that similar observations on the relative performance of ICO, CCO and MOCO were made when we performed the *in silico* leave-one-out cross-validation on the set of 27 high-expression genes of *E. coli* reported in an earlier study [33]. Details of this analysis can be found in Additional file 2.

## Discussion

### Capturing the preferred codon usage patterns

Earlier codon optimization studies have recommended the usage of high expression genes to design the recombinant gene for efficient heterologous expression [12,13,34]. In the analysis of codon usage patterns, the significant distinction in the ICU and CC distributions between highly expressed and other genes corroborated the relevance of identifying high-expression genes to characterize the preferred codon usage patterns. It is noted that although there is codon usage information readily available in the Codon Usage Database (http://www.kazusa.or.jp/codon/) [35], these data may not be useful as prior filtering of highly expressed genes was not performed. Such codon usage data may reflect some degree of preference for “rare” codons, thus leading to low gene expression [36].

Several options are available for quantifying the codon usage patterns. In this study, we have adopted the method of treating the ICU and CC distributions as a vector of frequency values to capture the relative abundance of individual codons and codon pairs. An earlier well-known method for quantifying codon usage bias is the codon adaptation index (CAI). The CAI has been widely used for codon optimization due to its observed correlation with gene expressivity [34]. However, by designing a gene through the maximization of CAI, the resultant coding sequence will become a “one amino acid – one codon” design where CAI = 1.0. This sequence design may not be desirable as the overexpression of this gene can lead to very rapid depletion of the specific cognate tRNAs resulting in tRNA pool imbalance, which can in turn cause an increase in translational errors [37]. In this aspect, the ICU fitness measure will be a better performance criterion than CAI since the former allows a small number of rare codons to be included in the final sequence. Furthermore, the calculation of CAI, as described in its original paper [34], is intrinsically based on individual codon usage and does not have the capability to account for codon pairing. Therefore, the information captured by the CC fitness cannot be reflected in the CAI value.

Therefore, the proposed approach of optimizing codons according to the complete ICU and CC distributions of highly expressed genes will be suitable to alleviate the problem of tRNA pool imbalance when the cell is induced to overexpress the target gene. As such, the concept of CAI was not considered in this study as this single value does not capture the details in ICU and CC distributions.

### Other potential issues in efficacy of CCO

Codon usage has been shown to affect the accuracy and speed of translation [38,39]. Hence, the concept of CCO implementation is to identify favorable codon pairings that can lead to more efficient protein synthesis process. Notably, an optimization framework based on the dynamic modeling of protein translation has been recently developed to identify suitable codon placements to improve translation elongation speed [40]. Although this method provides a mechanistic understanding of how codon choice affects translation efficiency, it requires a protein translation kinetic model and codon-specific elongation rates which may not be readily available for organisms other than *E. coli* as shown in previous studies [32,41]. Therefore, CCO may be a better alternative as it can achieve the aim of enhancing translation efficiency while having the advantage of utilizing information, including genome sequence and gene expression data, which are easily accessible in public databases such as the Gene Expression Omnibus (http://www.ncbi.nlm.nih.gov/geo/) and GenBank (http://www.ncbi.nlm.nih.gov/genbank/). Incidentally, there was evidence suggesting that translation initiation rather than elongation is the rate limiting step [42]. Nonetheless, CCO generated sequences can indirectly increase translation initiation by freeing up more ribosomes through enhanced translation elongation rates. The increased pool of free ribosomes can then help to improve translation initiation by mass action effect.

On the other hand, translation initiation can also be affected by the mRNA structure of the initiation site. At the primary structure level, Shine-Dalgarno sequence and Kozak sequence should be added to the 5’ end of the coding sequence since previous studies have shown that they are required for recognition of the AUG start codon to initiate translation in prokaryotes and eukaryotes, respectively, [43]. At the secondary structure level, it was found that hairpin, stem-loop and pseudoknot mRNA structures can repress protein translation [44]. Although this suggests that the computationally intensive mRNA secondary structure evaluation may be required for designing synthetic genes, it was also reported that the helicase activity of ribosome is able to disrupt the secondary structures for mRNA translation [45]. Therefore, we suggest using the mRNA secondary structure analysis only as a supplementary step for the CC-optimized sequences such that no significant computational cost is added to the main CCO procedure.

### CCO tool for synthetic biology

To further develop CCO into a software tool for designing synthetic genes, several other factors may have to be considered. From the experimental aspect, the gene optimization should take into consideration the types of restriction enzymes used for vector construction such that the restriction sites DNA motifs are avoided to prevent unnecessary cleavage of the coding sequence. In certain cases where the optimized coding sequence tends to have nucleotide repeats, additional steps may be required to avoid the repeats or inverted repeats which may lead to DNA recombination or formation of mRNA hairpin loops, respectively, that will reduce the heterologous expressivity of the target protein [46,47]. In addition, sequence homology may also be considered to design genes that are resistant to RNA interference such that complementary sequences of the silencing RNAs are avoided in the coding sequence [48]. Possible strategies to tackle the aforementioned issues during gene optimization have been discussed in a previous study [20].

The optimal sequences generated by CCO are not found in any natural organism. Thus, the CCO software tool should also consider challenges involved in the synthesis of these artificial genes. The current technology for *de novo* gene synthesis involves the chemical synthesis of short oligonucleotides followed by ligation- or PCR-mediated assembly of the oligonucleotides to form the complete gene [49]. The way in which a long coding sequence is broken down into short oligonucleotides has to be properly designed to minimize the oligonucleotide synthesis error rate and maximize the uniformity of the oligonucleotides’ annealing temperatures for efficient assembly. Several methods such as DNAWorks [50], Gene2Oligo [51] and TmPrime [52] have been proposed to these goals in oligonucleotide design optimization for gene synthesis. Although these oligonucleotide optimization methods can be performed independently from the codon optimization procedure, these two processes can be integrated to facilitate the “design-to-synthesis” workflow. As long as the current gene synthesis paradigm prevails, researchers can further explore the possibility of developing an integrated codon and oligonucleotide optimization software tool to effectively and systematically design high performance synthetic genes for protein expression.

### Potential applications of CCO

The motivation behind codon optimization is usually to enhance the expression of foreign genes in expression hosts such as *E. coli*, *P. pastoris* and *S.cerevisiae*. In addition, codon optimization can also be used to generate synthetic designs of native genes for metabolic engineering applications. While conventional overexpression of native metabolic genes is achieved by increasing gene copy number through the introduction of plasmids, codon optimization provides an alternative approach for enhancing pathway utilization via insertion of high-expression synthetic genes of the respective metabolic enzymes into the host’s genome. The latter technique can be advantageous as it obviates the metabolic burden associated with plasmid maintenance [53-55], thus allowing the cells to have more resources for growth and biochemical production.

Apart from biotechnological applications, codon optimization can also be used in biomedical research where modulation of protein expression is required to alter physiological response. For example, in the development of vaccines against viruses, one approach is to genetically manipulate the virus to obtain a “live attenuated” strain as the vaccine. Such a vaccine, when administered to the host, will elicit an immune response for the host to develop immunologic memory and specific immunity against the virus without severe disruption to the overall physiology. Some conventional methods of developing live attenuated vaccines include laboratory adaptation of virus in non-human hosts and random/site-directed mutagenesis [56]. Since the wild-type virus is able to hijack the gene expression machinery of the host for replication, the de-optimization of viral codon usage can lead to the development of live attenuated vaccines as demonstrated in a recent study [19]. Therefore, the CCO framework developed in this study can be slightly modified to design synthetic virus consisting of more rare codons that can be used as vaccines. Specifically, we can either invert the objective function to minimize CC fitness or alter the target CC distribution during the execution of the optimization procedure to design the sequence of the attenuated virus.

## Conclusions

Through novel implementations of ICO, CCO and MOCO, the high-expression genes of four microbial hosts were optimized and cross-validated to compare the performance of the optimized sequences. Amongst all the optimization approaches, CCO was found to generate the sequences that are most similar to the native high-expression genes, indicating a greater potential for high *in vivo* protein expression. Contrary to the conventional practice which adopts ICU optimization as the key element of gene design, our study suggests that CC fitness is a more relevant design parameter for optimizing the sequence for improved heterologous protein expression. Thus, future works to incorporate the optimization of CC fitness into synthetic gene design software can lead to the development of more efficient platforms for gene optimization.

## Methods

### Identifying highly expressed genes

Provided that highly expressed genes have evolved to adopt optimal codon patterns, information on ICU and CC preference of any organism can be extracted from the DNA sequences of the high-expression genes. In this sense, we used published microarray data of *E. coli*[57], *L. lactis*[58], *P. pastoris*[59] and *S. cerevisiae*[60] from various experimental conditions to identify the top 5% of genes with the highest expression value for each microbe. The ICU and CC of these genes were then extracted from their corresponding DNA coding sequences that can be obtained from publicly available genome annotations for *E. coli*[61], *L. lactis*[62], *P. pastoris*[63] and *S. cerevisiae*[64]. Each host’s ICU and CC preference can be represented as the frequency of occurrence of individual codons and codon pairs found in the sequences of the highly expressed genes. These ICU and CC distributions are then be used as the targets for the respective codon optimization methods. For the evaluation of ICU and CC biasness difference between high- and low-expression genes, the low-expression genes are identified in a similar way whereby the bottom 5% of the genes with the lowest expression values are consolidated (see Additional file 1 for a list of high-expression genes).

### ICU and CC biasness

To compute the significance of codon (pair) usage bias, we resort to the Pearson’s chi-squared test. Based on the null hypothesis that “the ICU (CC) of high-expression genes follows the uniform/unbiased distribution”, the chi-square statistic for amino acid (pair) *j* is calculated as:

(1) $${Χ}_{j}^{2}=\sum_{i=1}^{n_{j}^{H}}\frac{{\left(O_{\mathrm{ij}}^{H}-E_{\mathrm{ij}}^{H}\right)}^{2}}{E_{\mathrm{ij}}^{H}}$$

where *E*_*ij*_^*H*^ and *O*_*ij*_^*H*^ are the expected and observed numbers of synonymous codon (pair) *i* encoding amino acid (pair) *j*, respectively. The constant *n*_*j*_^*H*^ refers to the number of unique synonymous codon (pair) encoding the amino acid *j*; for example, the *n*_*j*_^*H*^ values for asparagine, glycine and leucine are 2, 4, and 6 respectively. The superscript “*H*” indicates that only the high-expression genes are used to evaluate the respective values. Given the null hypothesis of unbiased codon (pair) usage, *E*_*ij*_^*H*^ can be calculated as $E_{\mathrm{ij}}^{H}=\frac{N_{j}^{H}}{n_{j}^{H}}$ where *N*_*j*_^*H*^ refers to the total number of amino acid (pair) *j* found in the high-expression genes. The p-value is then evaluated by comparing the calculated *Χ*_*j*_^2^ against the *χ*^2^ distribution with (*n*_*j*_^*H*^ − 1) degrees of freedom since the reduction in degrees of freedom is one due to the constraint: $\sum_{i=1}^{n_{j}^{H}}O_{\mathrm{ij}}^{H}=N_{j}^{H}$. Using a p-value cut-off of 0.05, we can identify the amino acid (pair) with biased ICU (CC) distribution that is significantly different from the normal distribution. This test of ICU and CC biasness will be referred to as “*χ*^2^ Test 1”. To ensure that the statistical adequacy of this chi-squared test, any amino acid (pair) with low expected occurrence (i.e. *E*_*ij*_^*H*^ < 5) will be omitted from this analysis as recommended in an earlier study [65]. Furthermore, chi-squared test of singular amino acids (methionine and tryptophan) and amino acid pairs (pairs only consisting of methionine and/or tryptophan) are also not relevant since they are not encoded by more than one synonymous codon (pair) such that the chi-squared statistic will always be equal to 1.

The presented Pearson’s chi-squared formulation is slightly modified to determine whether the ICU (CC) is significantly different between high-expression genes and all genes in the genome. Based on the null hypothesis as “ICU (CC) of high-expression genes is the same as that of all genes in the genome”, the expected number of codon (pair) *i* in high-expression genes is modified as:

(2) $${\tilde{E}}_{\mathrm{ij}}^{H}=\frac{O_{\mathrm{ij}}^{A}N_{j}^{H}}{N_{j}^{A}}$$

where *O*_*ij*_^*A*^ refers the observed number of codon (pair) *i* encoding amino acid (pair) *j* and *N*_*j*_^*A*^ refers to the total number of amino acid (pair) *j*. The superscript “*A*” indicates that all genes in the host’s genome are used for evaluating the respective values. By substituting *E*_*ij*_^*H*^ with ${\tilde{E}}_{\mathrm{ij}}^{H}$ in the expression for *Χ*_*j*_^2^, the chi-squared statistic to test the difference in ICU (CC) distribution between high-expression genes and all genes in the host’s genome can be calculated.

### ICU and CC fitness evaluation

In this study, the target gene, subsequently known as the “subject”, is optimized such that the final synthetic sequence design will exhibit ICU and/or CC distributions that are as similar as possible to those preferred by the host’s organism. The ICU and CC fitness values can be used to quantify the degree of similarity in ICU and CC distributions between the subject and the host. Before formulating the ICU and CC fitness, we present the mathematical expression of the coding sequence and amino acid sequence as follows:

(3) $$\begin{array}{l} S_{\text{A,}1}=\left\{M,R,F,P,S,I,F,\ldots ,G,D,R,*\right\}\\ \;={\left\{\tau_{i,1}\right\}}_{i=1}^{n} \end{array}$$

(4) $$\begin{array}{l} S_{\text{C,}1}=\{AUG,AGA,UUU,CCU,UCA,\ldots ,GAC,\\ \;AGA,UGA\}={\left\{\lambda_{i,1}\right\}}_{i=1}^{n} \end{array}$$

(5) $$\tau_{i,1}\in Α={\left\{\alpha^{j}\right\}}_{j=1}^{21}=\left\{A,C,D,\ldots ,W,Y,*\right\}\;\forall i$$

(6) $$\begin{array}{l} \lambda_{i,1}\in Κ={\left\{\kappa^{k}\right\}}_{k=1}^{64}\\ \;=\left\{AAA,AAC,AAG,\ldots ,UUG,UUU\right\}\;\forall i \end{array}$$

where *τ*_*i*,1_ refers to the amino acid occupying the *i*^th^ position of the amino acid sequence *S*_A,1_ with the subscript 1 indicating the target protein; *τ*_*i*,1_ also belongs to the set A of 21 unique amino acids *α*^*j*^. Similarly, *λ*_*i*,1_, a codon from the set K of 64 unique codons *κ*^*k*^, represents the codon variable in the *i*^th^ position of the target coding sequence *S*_C,1_. It is noted that the coding sequence is express as a sequence of codons instead of nucleotides since codon usage patterns is the key concern. As codon context is another key issue to be examined, we also include the following mathematical expressions for amino acid pairs and codon pairs:

(7) $$\begin{array}{l} S_{\text{AA,}1}=\left\{MR,RF,FP,PS,SI,,\ldots ,GD,DR,R*\right\}\\ \;={\left\{\omega_{i,1}\right\}}_{i=1}^{n-1} \end{array}$$

(8) $$\begin{array}{l} S_{\text{CC,}1}=\{AUGAGA,AGAUUU,UUUCCU,\ldots ,\\ \;AGAUGA\}={\left\{\gamma_{i,1}\right\}}_{i=1}^{n-1} \end{array}$$

(9) $$\begin{array}{l} \omega_{i,1}\in Β=\left\{AA,AC,CA,\ldots ,W*,Y*\right\}\\ \;={\left\{\beta^{j}\right\}}_{j=1}^{420}\;\forall i\in \left\{1,\ldots ,n-1\right\} \end{array}$$

(10) $$\begin{array}{l} \gamma_{i,1}\in Ρ=\left\{AAAAAA,\ldots ,UUUUUU\right\}\\ \;={\left\{\rho^{k}\right\}}_{k=1}^{3904}\;\forall i\in \left\{1,\ldots ,n-1\right\} \end{array}$$

By defining a function to *f* translate codon(s) to the corresponding amino acid(s) and a concatenation function *g*(*a*,*b*) to append the string *b* to right of string *a*, we have the following mathematical relationships for *τ*_*i*,1_, *ω*_*i*,1_, *λ*_*i*,1_ and *γ*_*i*,1_:

(11) $$f\left(\lambda_{i,1}\right)=\tau_{i,1}$$

(12) $$f\left(\gamma_{i,1}\right)=\omega_{i,1}$$

(13) $$g\left(\tau_{i,1},\tau_{\left(i+1\right),1}\right)=\omega_{i,1}$$

(14) $$g\left(\lambda_{i,1},\lambda_{\left(i+1\right),1}\right)=\gamma_{i,1}$$

The ICU distribution can be defined as the frequency of each unique codon based on its total number of occurrences in the sequence(s). Based on the mathematical formulation presented hitherto, the required mathematical expressions to calculate the ICU distribution are as follows:

(15) $$\theta_{\text{A,}1}^{j}=\sum_{i=1}^{n}1\left\{\tau_{i,1}=\alpha^{j}\right\}\;\forall j\in \left\{1,2,\ldots ,21\right\}$$

(16) $$\theta_{\text{C,}1}^{k}=\sum_{i=1}^{n}1\left\{\lambda_{i,1}=\kappa^{k}\right\}\;\forall k\in \left\{1,2,\ldots ,64\right\}$$

(17) $$\theta_{\text{A,}0}^{j}=\sum_{i=1}^{n^{\prime }}1\left\{\tau_{i,0}=\alpha^{j}\right\}\;\forall j\in \left\{1,2,\ldots ,21\right\}$$

(18) $$\theta_{\text{C,}0}^{k}=\sum_{i=1}^{n^{\prime }}1\left\{\lambda_{i,0}=\kappa^{k}\right\}\;\forall k\in \left\{1,2,\ldots ,64\right\}$$

(19) $$p_{0}^{k}=\frac{\theta_{\text{C,}0}^{k}}{\sum_{j=1}^{21}\left[\theta_{\text{A,}0}^{j}\times 1\left\{\alpha^{j}=f\left(\kappa^{k}\right)\right\}\right]}\;\forall k\in \left\{1,2,\ldots ,64\right\}$$

(20) $$p_{1}^{k}=\frac{\theta_{\text{C,}1}^{k}}{\sum_{j=1}^{21}\left[\theta_{\text{A,}1}^{j}\times 1\left\{\alpha^{j}=f\left(\kappa^{k}\right)\right\}\right]}\;\forall k\in \left\{1,2,\ldots ,64\right\}$$

(21) $$\theta_{\text{C,}0}^{k}=\sum_{i=1}^{n^{\prime }}1\left\{\lambda_{i,0}=\kappa^{k}\right\}\;\forall k\in \left\{1,2,\ldots ,64\right\}$$

(22) $$\theta_{\text{C,}0}^{k}=\sum_{i=1}^{n^{\prime }}1\left\{\lambda_{i,0}=\kappa^{k}\right\}\;\forall k\in \left\{1,2,\ldots ,64\right\}$$

where 1{·} is an indicator function such that $1\left\{x\right\}=\{\begin{array}{l} 1\;\text{if}\;x\;\text{is true}\\ 0\;\text{otherwise} \end{array}$.

The count variables *θ*_AA_^*j*^ and *θ*_C_^*k*^ refer to the numbers of occurrences of amino acid *j* and codon *k*, respectively, found in the host’s (indicated by subscript “0”) or subject’s (indicated by subscript “1”) sequence(s), while *p*^*k*^ represents the frequency of occurrence of codon *k*. Accordingly, the ICU fitness can be expressed as:

(23) $$\Psi_{\text{ICU}}=-\frac{\sum_{k=1}^{64}\left|p_{0}^{k}-p_{1}^{k}\right|}{64}$$

The ICU fitness, *Ψ*_ICU_, was divided by 64 such that the numerical value will reflect the average fitness of all codons. In a similar way, if we denote the frequency of occurrence of codon pair *k* as *q*^*k*^, the CC fitness can be calculated as:

(24) $$q_{0}^{k}=\frac{\theta_{\text{CC,}0}^{k}}{\sum_{j=1}^{420}\left[\theta_{\text{AA,}0}^{j}\times 1\left\{\beta^{j}=f\left(\rho^{k}\right)\right\}\right]}\;\forall k\in \left\{1,2,\ldots ,3904\right\}$$

(25) $$q_{1}^{k}=\frac{\theta_{\text{CC,}1}^{k}}{\sum_{j=1}^{420}\left[\theta_{\text{AA,}1}^{j}\times 1\left\{\beta^{j}=f\left(\rho^{k}\right)\right\}\right]}\;\forall k\in \left\{1,2,\ldots ,3904\right\}$$

(26) $$\Psi_{\text{CC}}=-\frac{\sum_{k=1}^{3904}\left|q_{0}^{k}-q_{1}^{k}\right|}{3904}$$

(See Additional file 3 for further details of the mathematical formulation).

## Competing interests

The authors declare that they have no competing interests.

## Authors’ contributions

BKSC and DL conceived the codon optimization idea. BKSC developed the algorithm, performed the computational simulations and drafted the manuscript. DL revised the manuscript. Both authors read and approved the final manuscript.

## Supplementary Material

Additional file 1 **List of Sequences.** Excel file contains DNA sequences of wild-type high- and low-expression genes; the optimized genes generated by the *in silico* leave-one-out cross validation are also included. Click here for file

Additional file 2 **Codon optimization of another set of high-expression genes in*E. coli*.** ICO, CCO and MOCO were carried out using the set of high-expression genes reported in an earlier study [33] to evaluate the relative performance of the methods. Click here for file

Additional file 3 **Formulation of codon optimization methods.** Detailed mathematical formulation of ICO, CCO and MOCO. Click here for file
