# Supplementary material for: Computational codon optimization of synthetic gene for protein expression
Source: BMC Syst Biol. 2012 Oct 20;6:134. doi: 10.1186/1752-0509-6-134 (PMC3495653; doi:10.1186/1752-0509-6-134)
Supplement: Additional file 2 — Codon optimization of another set of high-expression genes inE. coli. ICO, CCO and MOCO were carried out using the set of high-expression genes reported in an earlier study [33] to evaluate the relative performance of the methods. [file 1752-0509-6-134-S2.doc]

# Codon optimization of another set of high-expression genes in *E. coli*

A list of 27 high-expression genes (Table S3.1) has been used to establish a correlation between codon usage bias and gene expression in previous studies . Using these genes, we performed the *in silico* leave-one-out cross validation to evaluate the performance of ICO, CCO and MOCO methods. Results showed that CCO generally produces sequences that best matches the wild-type highly expressed sequences, followed by the MOCO and ICO methods (Figure S3.1). The optimized sequences are compared pairwise in a tournament style based on their percentage of matching codons with the respective wild-type sequences to generate the tournament matrix (Table 3.2). It was observed that CCO performed better than ICO and MOCO in at least 70 % of the instances. This result is consistent with that presented in the main manuscript, indicating that CC fitness is a more important design criterion than ICU fitness for gene optimization.

# References

1. Sharp PM, Li WH: **Codon usage in regulatory genes in Escherichia coli does not reflect selection for 'rare' codons.** *Nucleic Acids Res* 1986, **14:**7737-7749.

2. Sharp PM, Li WH: **The codon adaptation index--a measure of directional synonymous codon usage bias, and its potential applications.** *Nucleic Acids Res* 1987, **15:**1281-1295.

# Figures

**
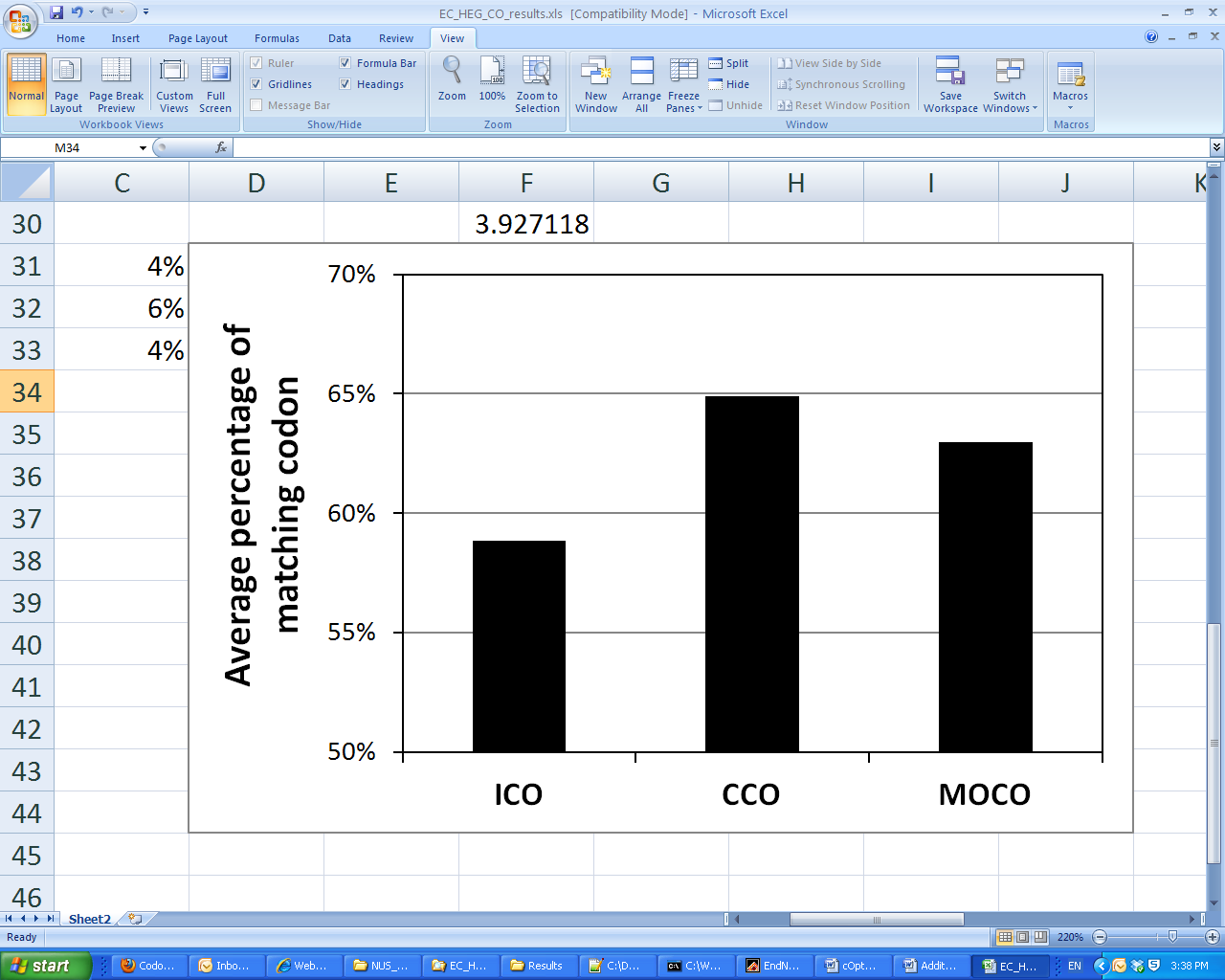
**

**Figure S3.1.** Comparison of performance of codon optimization methods.

# Tables

Table S3.1. List of high-expression genes.

| **Gene** | **Locus tag** | **Product** |
| --- | --- | --- |
| rpsU | b3065 | 30S ribosomal subunit protein S21 |
| rpsJ | b3321 | 30S ribosomal subunit protein S10 |
| rpsL | b3342 | 30S ribosomal subunit protein S12 |
| rpsT | b0023 | 30S ribosomal subunit protein S20 |
| rpsA | b0911 | 30S ribosomal subunit protein S1 |
| rpsB | b0169 | 30S ribosomal subunit protein S2 |
| rpsO | b3165 | 30S ribosomal subunit protein S15 |
| rpsG | b3341 | 30S ribosomal subunit protein S7 |
| rpmB | b3637 | 50S ribosomal subunit protein L28 |
| rpmG | b3636 | 50S ribosomal subunit protein L33 |
| rpmH | b3703 | 50S ribosomal subunit protein L34 |
| rplK | b3983 | 50S ribosomal subunit protein L11 |
| rplJ | b3985 | 50S ribosomal subunit protein L10 |
| rplA | b3984 | 50S ribosomal subunit protein L1 |
| rplL | b3986 | 50S ribosomal subunit protein L7/L12 |
| rplQ | b3294 | 50S ribosomal subunit protein L17 |
| rplC | b3320 | 50S ribosomal subunit protein L3 |
| lpp | b1677 | murein lipoprotein |
| ompA | b0957 | outer membrane protein A (3a;II*;G;d) |
| ompC | b2215 | outer membrane porin protein C |
| ompF | b0929 | outer membrane porin 1a (Ia;b;F) |
| tufA | b3339 | protein chain elongation factor EF-Tu (duplicate of tufB) |
| tufB | b3980 | protein chain elongation factor EF-Tu (duplicate of tufA) |
| tsf | b0170 | protein chain elongation factor EF-Ts |
| fusA | b3340 | protein chain elongation factor EF-G, GTP-binding |
| recA | b2699 | DNA strand exchange and recombination protein with protease and nuclease activity |
| dnaK | b0014 | chaperone Hsp70, co-chaperone with DnaJ |

Table S3.2. Tournament matrix.

|  | **ICO** | **CCO** | **MOCO** |
| --- | --- | --- | --- |
| **ICO** |  | 3 | 2 |
| **CCO** | 23 |  | 19 |
| **MOCO** | 25 | 7 |  |

Each cell indicates the number of wins by the method in the leftmost column over a total of 27 tournaments. Whenever the numbers of wins and losses (i.e. cells diagonally opposite of each other) do not sum up to 27, the shortfall will be equal to the number of draws.
