# Supplementary material for: Computational codon optimization of synthetic gene for protein expression
Source: BMC Syst Biol. 2012 Oct 20;6:134. doi: 10.1186/1752-0509-6-134 (PMC3495653; doi:10.1186/1752-0509-6-134)
Supplement: Additional file 3 — Formulation of codon optimization methods. Detailed mathematical formulation of ICO, CCO and MOCO. [file 1752-0509-6-134-S3.doc]

# Codon optimization mathematical formulation

The amino acid sequence of the recombinant EPO sequence with MFα signal peptide as the target protein for heterologous expression in *P. pastoris* is as follows:

MRFPSIFTAVLFAASSALAAPVNTTTEDETAQIPAEAVIGYLDLEGDFDVAVLPFSNSTNNGLLFINTTIASIAAKEEGVSLDKRAPPRLICDSRVLERYLLEAKEAENITTGCAEHCSLNENITVPDTKVNFYAWKRMEVGQQAVEVWQGLALLSEAVLRGQALLVNSSQPWEPLQLHVDKAVSGLRSLTTLLRALGAQKEAISPPDAASAAPLRTITADTFRKLFRVYSNFLRGKLKLYTGEACRTGDR*

The abbreviation and corresponding synonymous codons for each amino acid is shown in Table S1.1.

Based on the recombinant EPO sequence, we illustrate the following mathematical formulation of the codon optimization problem.

## Mathematical representation of RNA and protein sequences

The primary structure of the target protein can be described as a sequence amino acids mathematically denoted as:

where $\tau_{i,1}$ refers to the amino acid occupying the $i^{\text{th}}$ position of the protein sequence $S_{\text{A,1}}$ with the subscript 1 indicating that this is the target protein and $n$ refers to the sequence length which is 252 for the recombinant EPO. Each $\tau_{i,1}$ belongs to the set of unique amino acids which also includes the translation termination signal. Therefore, the following relationship can be established:

Since the primary concern is the manipulation of codons, the nucleotide sequence will be defined in terms of nucleotide triplets instead of individual nucleotides. Therefore, the coding sequence of the EPO gene will be mathematically written as:

where $\lambda_{i,1}$ refers to the codon variable in the $i^{\text{th}}$ position of the target coding sequence $S_{C}$. Every variable $\lambda_{i,1}$ belongs to the set of 64 unique codons such that:

By defining a function $f$ to many any codon to its corresponding amino acid sequence, the translation of mRNA to protein can be written as for individual codons, or for the entire coding sequence. In ICU optimization, every $\lambda_{i,1}$ is a variable while $\tau_{i,1}$ is a predefined constant. Therefore, the constraint delineates the feasible solution space of the ICU optimization problem. It is noted that in this writing, the subscript will be consistently used to indicate the position in a sequence while a superscript will always be used as index for elements in a unique set.

## ICU fitness

After defining the variables (codons) and constraint (target protein sequence), the final component of the optimization problem formulation is the objective function. In ICU optimization, the aim is to search for a candidate coding sequence which exhibits an ICU pattern that is most similar to the host’s. Therefore, a fitness measure can be used to quantify the similarity between the ICU distributions of the host and the designed coding sequence, subsequently known as the “subject”.

The ICU distribution can be mathematically written as a vector of individual codon frequencies. The frequency of a codon can be calculated by dividing the number of codon occurrences in a coding sequence by the total number of corresponding amino acid occurrences in the target protein sequence. The counts of codons and amino acids are mathematically formulated as follows:

Subject’s count for amino acid $j$:

Subject’s count for codon $k$:

Host’s count for amino acid $j$:

Host’s count for codon $k$:

where is an indicator function such that

It is noted that the host’s codon and amino acid counts are calculated for a group of selected native genes while the subject’s counts are calculated for the target protein sequence only. Hence, the host’s counts are summed over the total number of amino acids/codons in all the genes denoted by $n^{'}$. Accordingly, the subject’s codon frequency can be calculated as:

And the corresponding host’s codon frequency can be calculated as:

The ICU distributions can be written as vectors of 64 ICU frequencies, i.e. $p_{0}$ and $p_{1}$. Thus, the ICU fitness of the subject with respect to the host can be expressed as the negative of the Manhattan distance between $p_{0}$ and $p_{1}$:

## Individual codon optimization (ICO)

By combining the mathematical expressions presented thus far, the ICU optimization problem can be formulated as follows:

(P1) max $Z=\Psi_{\text{ICU}}$

s.t.

Due to the discrete codon variables and nonlinear fitness expression of $\Psi_{\text{ICU}}$, the above is a mixed-integer nonlinear programming (MINLP) problem. Nonetheless, the problem can be linearized using a similar strategy shown earlier in Error: Reference source not found. By decomposing the nonlinear expression into a series of linear constraints which consist of positive real and integer variables, the MINLP problem (P1) can be recast into a MILP problem . Although such an optimization problem can be solved using MILP solvers, there is a faster method for generating a subject with optimal ICU using the following steps:

1. Calculate the host’s individual codon usage distribution, $p_{0}^{k}$.
2. Calculate the subject’s amino acid counts, $\theta_{\text{A,1}}^{j}$.
3. Calculate the optimal codon counts for the subject:
4. For each $\tau_{i}$ in the subject’s sequence, randomly assign a codon $\kappa^{k}$ if $\theta_{C}^{k}>0$, and decrement by one.
5. Repeat step 4 for all amino acids of the target protein from $\tau_{1,1}$ to $\tau_{n,1}$.

## Codon context optimization (CCO)

The formulation of CC optimization is similar to that of ICU optimization. In the context of CC, the target coding sequence is expressed as a sequence of codon pair variables:

where $\gamma_{i,1}$ refers to the codon variable in the $i^{\text{th}}$ position of the target coding sequence $S_{\text{CC}}$. It is noted that the sequence $S_{\text{CC}}$ is different from sequence $S_{C}$ as the former set consists of $n-1$ codon pairs while the latter is made up of $n$ codons. By defining a concatenation function $g\left( a,b \right)$ to append the string $b$ to right of string $a$, the relationship between $\lambda_{i,1}$ and $\gamma_{i,1}$ can be stated as . Every codon pair variable encodes for the corresponding amino acid pair, i.e. , and they each belong to the unique sets of amino acid pairs and codon pairs defined as follows:

Therefore, the counts and frequency can be expressed as follows:

Subject’s count for amino acid pair $j$:

Subject’s count for codon pair $k$:

Subject’s frequency of codon pair $k$:

Host’s count for amino acid pair $j$:

Host’s count for codon pair $k$:

Host’s frequency of codon pair $k$:

By denoting the CC distributions of the host and the subject as $q_{0}$ and $q_{1}$, the CC fitness of the subject is expressed as:

Consequently, the mathematical formulation of the CC optimization problem is as follows:

(P2) max $Z=\Psi_{\text{CC}}$

s.t.

The above MINLP problem can also be recast into an MILP problem using the same strategy shown earlier in ICO to harness the more efficient MILP solvers to find an optimal sequence design. Due to the large discrete nonlinear search space, existing MILP solvers which use either branch-and-bound or branch-and-cut methods will still require huge amount of computational resources to find the optimum solution . Therefore, the genetic algorithm is used to solve (P2) as it provides an intuitive framework whereby codons are “evolved” towards optimal CC through techniques mimicking natural evolutionary processes such as selection, crossover or recombination and mutation.

The steps involved in the implementation of genetic algorithm for CC optimization is as follows:

1. Randomly initialize a population of coding sequences for target protein.
2. Evaluate the CC fitness of each sequence in the population.
3. Rank the sequences by CC fitness and check termination criterion.
4. If termination criterion is not satisfied, select the “fittest” sequences (top 50% of the population) as the parents for creation of offsprings via recombination and mutation.
5. Combine the parents and offsprings to form a new population.
6. Repeat steps 2 to 5 until termination criterion is satisfied.

In step 3, the termination criterion depends on the degree of improvement in best CC fitness values for consecutive generations of the genetic algorithm. If the improvement in CC fitness across many generations is not significant, the algorithm is said to have converged. In this study, the CC optimization algorithm will terminate when there is less than 0.5% increase in CC fitness across 100 generations, i.e. where $r$ refers to the $r^{\text{th}}$ generation of the genetic algorithm. When the termination criterion is not satisfied, the subsequent step 4 will perform an elitist selection such that the fittest 50 % of the population are always selected for reproduction of offsprings through recombination and mutation. During recombination, a pair of parents is chosen at random and a crossover is carried out at a randomly selected position in the parents’ sequences to create 2 new individuals as offsprings. The offsprings subsequently undergo a random point mutation before they are combined with the parents to form the new generation.

Unlike traditional implementations of genetic algorithm where individuals in the population are represented as as 0-1 bit strings, the presented CC optimization algorithm represents each individual as a sequential list of character triplets indicating the respective codons. Therefore, the codons can be manipulated directly with reference to a hash table which defines the synonymous codons for each amino acid. As a result, the protein encoded by the coding sequences is always the same in the genetic algorithm since crossovers only occur at the boundary of the codon triplets and mutation is always performed with reference to the hash table of synonymous codons for each respective amino acid. The hash table is constructed according to Table S1.1.


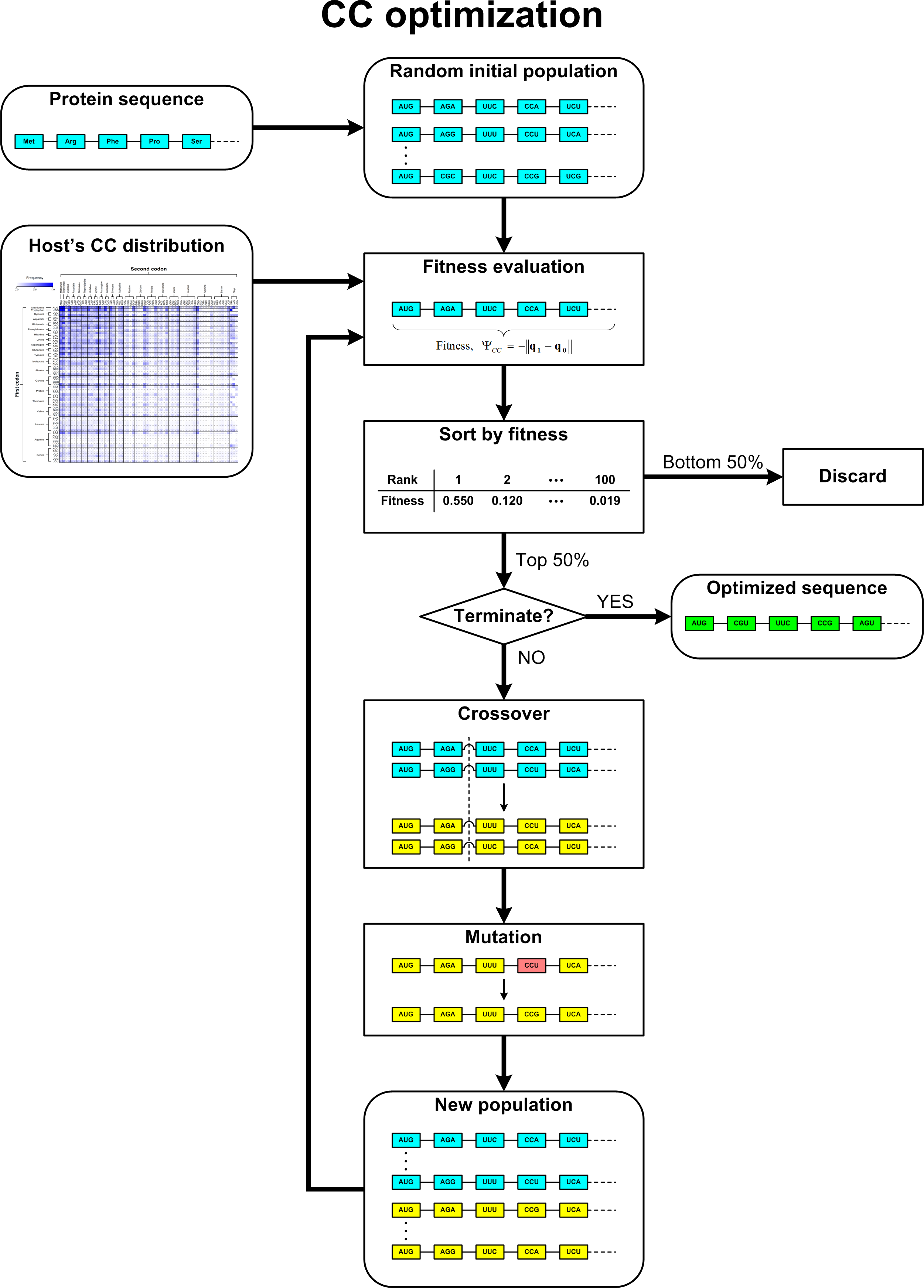


## Multi-objective codon optimization (MOCO)

Based on the formulations for ICU and CC optimization, the MOCO problem can be mathematically formulated as follows:

(P6) max

s.t.

Due to the complexity attributed to CC optimization, solution to (P6) will also require a heuristic method. In this case, the nondominated sorting genetic algorithm-II (NSGA-II) is used to solve the nonlinear multi-objective optimization problem . The procedure for NSGA-II is similar to that presented for CC optimization except for additional steps required to identify the nondominated solution sets and the ranking of these sets to identify the pareto optimum front. The NSGA-II procedure for solving the MOCO problem is as follows:

1. Randomly initialize a population of coding sequences for target protein.
2. Evaluate ICU and CC fitness of each sequence in the population.
3. Group the sequences into nondominated sets and rank the sets.
4. Check termination criterion.
5. If termination criterion is not satisfied, select the “fittest” sequences (top 50% of the population) as the parents for creation of offsprings via recombination and mutation.
6. Combine the parents and offsprings to form a new population.
7. Repeat steps 2 to 5 until termination criterion is satisfied.

The identification and ranking of nondominated sets in step 3 is performed via pair-wise comparison of the sequences’ ICU and CC fitness. For a given pair of sequences with fitness values expressed as and , the domination status can be evaluated as follows:

- If and , sequence 1 dominates sequence 2.
- If and , sequence 1 dominates sequence 2.
- If and , sequence 2 dominates sequence 1.
- If and , sequence 2 dominates sequence 1.

Whenever a particular sequence is found to be dominated by another sequence, the domination rank of the former sequence is lowered. As such, the grouping and sorting of the nondominated sets are performed simultaneously in step 3 using the pseudo code:

*Initialize domination ranks of all individuals to zero;*

*For every individual i where i loops from 1 to (n-1),*

*For every individual j where j loops from i to n,*

*If individual i dominates individual j,*

*Increment domination value of j;*

*Else if individual j dominates individual I,*

*Increment domination value of i;*

*Sort individuals based on domination ranks;*

In the original nondominated sorting algorithm , the set of individuals that is dominated by every individual is stored in memory. Therefore, for a total population of $n$, the total storage requirement is $O\left( n^{2} \right)$. However, for the abovementioned algorithm, only $O\left( n \right)$ storage is required for storing the domination value of each individual. In terms of computational complexity, both the original and modified algorithm requires at most computations for $m$ objective values since all the $n$ individuals have to be compared pair-wise for every objective to be optimized. Therefore, the nondominated sorting algorithm presented in this thesis is superior on the whole, especially with regards to computational storage requirement which can become an important issue when dealing with long coding sequences.

# References

1. Chung BK, Lee DY: **Flux-sum analysis: a metabolite-centric approach for understanding the metabolic network.** *BMC Syst Biol* 2009, **3:**117.

2. Atamtürk A, Savelsbergh M: **Integer-Programming Software Systems.** *Annals of Operations Research* 2005, **140:**67-124.

3. Goldberg DE: *Genetic algorithms in search, optimization, and machine learning.* Addison-Wesley Pub. Co.; 1989.

4. Deb K, Pratap A, Agarwal S, Meyarivan T: **A fast and elitist multiobjective genetic algorithm: NSGA-II.** *IEEE Trans Evo Comp* 2002, **6:**182-197.

# Tables

Table S1.1. Amino acid abbreviation and synonymous codons.

| **Amino acid** | **Abbreviation** | **Synonymous codon(s)** |
| --- | --- | --- |
| Methionine | M | AUG |
| Tryptophan | W | UGG |
| Cysteine | C | UGC, UGU |
| Aspartate | D | GAC, GAU |
| Glutamate | E | GAA, GAG |
| Phenylalanine | F | UUC, UUU |
| Histidine | H | CAC, CAU |
| Lysine | K | AAA, AAG |
| Asparagine | N | AAC, AAU |
| Glutamine | Q | CAA, CAG |
| Tyrosine | Y | UAC, UAU |
| Isoleucine | I | AUA, AUC, AUU |
| Alanine | A | GCA, GCC, GCG, GCU |
| Glycine | G | GGA, GGC, GGG, GGU |
| Proline | P | CCA, CCC, CCG, CCU |
| Threonine | T | ACA, ACC, ACG, ACU |
| Valine | V | GUA, GUC, GUG, GUU |
| Leucine | L | CUA, CUC, CUG, CUU, UUA, UUG |
| Arginine | R | AGA, AGG, CGA, CGC, CGG, CGU |
| Serine | S | AGC, AGU, UCA, UCG, UCC, UCU |
| (Stop) | * | UAA,UAG,UGA |
